# Correlation and clinical significance of GSTP1 hypermethylation in hepatocellular carcinoma: a systematic review and meta-analysis

**DOI:** 10.3389/fgene.2025.1543261

**Published:** 2025-02-20

**Authors:** Pengfei Li, Lei He, Chunxia Zhang, Xinyao Huang, Rong Sun, Yan Zhang, Yan Wang

**Affiliations:** ^1^ Department of Clinical Laboratory, Jiangsu Province Hospital of Chinese Medicine, Affiliated Hospital of Nanjing University of Chinese Medicine, Nanjing, China; ^2^ Second Outpatient Department, General Hospital of Eastern Theater Command, Nanjing, China; ^3^ State Key Laboratory of Supramolecular Structure and Materials, College of Chemistry, Jilin University, Changchun, China; ^4^ Institute of Theoretical Chemistry, College of Chemistry, Jilin University, Changchun, China; ^5^ First Clinical Medical College, Nanjing University of Chinese Medicine, Nanjing, China; ^6^ Department of Radiation Oncology, Jinling Hospital, Affiliated Hospital of Medical School, Nanjing University, Nanjing, China

**Keywords:** hepatocellular carcinoma, GSTP1, hypermethylation, biomarker, diagnosis, prognosis, meta-analysis

## Abstract

**Background:**

Hepatocellular carcinoma (HCC) is one of the most prevalent and fatal cancers globally, with poor prognosis due to late-stage diagnosis and limited early detection methods. GSTP1 gene hypermethylation has been implicated in various cancers, including HCC, as a potential biomarker for diagnosis, prognosis, and therapeutic strategies. This systematic review and meta-analysis aimed to assess the association between GSTP1 hypermethylation and HCC, and its clinical significance.

**Methods:**

A comprehensive literature search was conducted across PubMed, Embase, Web of Science, and the Cochrane Library to identify studies examining GSTP1 hypermethylation in HCC. Studies included in the meta-analysis were observational (case-control, cohort) or experimental studies (clinical trials) that reported on the correlation between GSTP1 hypermethylation and clinical outcomes in HCC patients. Pooled odds ratios (ORs) and weighted mean differences (WMDs) were calculated using random or fixed-effects models based on heterogeneity.

**Results:**

A total of 10 case-control studies were included, comprising 1,133 participants. The analysis revealed a significant association between GSTP1 hypermethylation and the presence of HCC (OR = 6.64, 95% CI: 2.17–20.38). GSTP1 hypermethylation was more frequently observed in liver cancer tissue compared to liver tissue from patients with other diseases (P < 0.00001). Additionally, a significant correlation between GSTP1 hypermethylation and poor clinical outcomes, such as advanced tumor stage, recurrence, and reduced overall survival, was observed (OR = 2.56, 95% CI: 1.80–3.64). Subgroup analyses based on study design, sample type, and detection method showed no significant heterogeneity in most comparisons.

**Conclusion:**

GSTP1 hypermethylation is significantly associated with the presence of HCC and poorer clinical outcomes, making it a promising biomarker for early diagnosis and prognosis. These findings highlight the potential for GSTP1 methylation as a diagnostic and prognostic tool in HCC management. Further large-scale, multicenter studies are required to standardize detection methods and evaluate the therapeutic potential of epigenetic reactivation of GSTP1 in HCC patients.

## 1 Background

Hepatocellular carcinoma (HCC) represents one of the most prevalent and deadly forms of cancer globally, contributing significantly to cancer-related mortality. Despite advances in therapeutic strategies, the prognosis for HCC patients remains poor due to late-stage diagnosis and the aggressive nature of the disease. As such, there is a pressing need for reliable biomarkers that can aid in early detection, prognosis, and personalized treatment of HCC. One such promising biomarker is the hypermethylation of the glutathione S-transferase pi 1 (GSTP1) gene (Boudal et al., 2012).

GSTP1 is a member of the glutathione S-transferase family, enzymes involved in the detoxification of a wide range of carcinogens, including reactive oxygen species and electrophilic compounds (Tchou et al., 2000). The GSTP1 gene is located on chromosome 11q13 and plays a critical role in cellular defense mechanisms against oxidative stress and xenobiotics (Cairns et al., 2001). The silencing of GSTP1 through promoter hypermethylation has been implicated in the pathogenesis of various cancers, including prostate, breast, and lung cancers (Gurioli et al., 2018). This epigenetic alteration results in the inactivation of GSTP1, leading to increased susceptibility to oxidative damage and mutagenesis, thereby contributing to carcinogenesis (Lee, 2007).

In HCC, the aberrant methylation of the GSTP1 promoter has been reported with varying frequencies, suggesting a potential role in liver carcinogenesis (Boudal et al., 2012). Numerous studies have investigated the relationship between GSTP1 hypermethylation and HCC, aiming to elucidate its diagnostic, prognostic, and therapeutic implications. However, the findings have been inconsistent, with some studies indicating a strong association between GSTP1 hypermethylation and HCC progression, while others have reported no significant correlation. This discrepancy underscores the need for a comprehensive synthesis of the existing evidence to better understand the clinical relevance of GSTP1 hypermethylation in HCC. The process of DNA methylation, particularly in the context of tumor suppressor genes like GSTP1, involves the addition of a methyl group to the cytosine residue of CpG islands in the promoter region. This epigenetic modification typically leads to transcriptional repression and subsequent gene silencing (Zhong et al., 2002a). In cancer, hypermethylation of tumor suppressor genes disrupts normal cellular regulatory mechanisms, thereby promoting oncogenesis. In the case of GSTP1 (Witt et al., 2022), its inactivation through hypermethylation can diminish the cell’s capacity to detoxify carcinogens, thus facilitating the accumulation of genetic damage and tumor development (Lasabova et al., 2010).

The clinical implications of GSTP1 hypermethylation in HCC are multifaceted. As a potential biomarker, GSTP1 methylation status could serve as an early indicator of malignant transformation in the liver (Haluskova et al., 2015). This is particularly important given the asymptomatic nature of early-stage HCC and the lack of effective screening methods. Additionally, assessing GSTP1 methylation could provide prognostic information, as some studies have suggested a correlation between hypermethylation and poor clinical outcomes, including advanced tumor stage, higher recurrence rates, and reduced overall survival.

Furthermore, the methylation status of GSTP1 may have therapeutic relevance. Epigenetic therapies, such as DNA methyltransferase inhibitors (e.g., azacitidine and decitabine), aim to reverse abnormal methylation patterns and restore the expression of silenced tumor suppressor genes. In HCC, such strategies could potentially reactivate GSTP1 and enhance the cellular defense against carcinogens, thereby inhibiting tumor growth and progression. However, the efficacy and safety of these therapies in the context of HCC require further investigation through well-designed clinical trials (Nakayama et al., 2004).

In addition to its role as a biomarker and therapeutic target, GSTP1 hypermethylation may also provide insights into the molecular mechanisms underlying HCC pathogenesis. Understanding the epigenetic regulation of GSTP1 and its interaction with other oncogenic pathways could reveal novel targets for intervention and contribute to the development of more effective treatment strategies (Meiers et al., 2007). Despite the potential significance of GSTP1 hypermethylation in HCC, several challenges and limitations exist in the current body of research. The heterogeneity of study designs, populations, and methodologies contributes to the variability in reported findings. Differences in sample size, tissue types (e.g., tumor tissue vs. adjacent non-tumor tissue), and methylation detection techniques (e.g., methylation-specific PCR, bisulfite sequencing) can affect the consistency and comparability of results. Additionally, the lack of standardization in defining hypermethylation thresholds further complicates the interpretation of data (Lou et al., 2008).

To address these gaps, our systematic review and meta-analysis aim to provide a robust synthesis of the existing literature on GSTP1 hypermethylation in HCC. By pooling data from multiple studies, we seek to quantify its association with HCC, evaluate its diagnostic and prognostic significance, and explore potential heterogeneity across studies. Additionally, our analysis contributes to advancing the field by offering insights into the clinical utility of GSTP1 hypermethylation as a biomarker and discussing its potential integration into diagnostic and therapeutic frameworks.

## 2 Method

### 2.1 Study design and registration

This systematic review and meta-analysis were conducted following the Preferred Reporting Items for Systematic Reviews and Meta-Analyses (PRISMA) guidelines.

### 2.2 Literature search strategy

A comprehensive literature search was conducted across PubMed, Embase, Web of Science, and the Cochrane Library from inception to 1 December 2024. Duplicates were identified and removed using reference management software (EndNote) prior to the screening of titles and abstracts. The search strategy included a combination of Medical Subject Headings (MeSH) and free-text terms related to “GSTP1,” “hypermethylation,” “methylation,” “hepatocellular carcinoma,” “liver cancer,” and “HCC.” Studies published in languages other than English were considered. Translation tools were employed to evaluate non-English studies to ensure inclusivity. No studies were excluded based on translation difficulties. Additionally, reference lists of relevant articles and reviews were manually screened to identify any additional studies.

Filters were applied during the study selection process to focus on observational studies (case-control, cohort) and experimental studies (clinical trials) that examined the relationship between GSTP1 hypermethylation and hepatocellular carcinoma (HCC). No restrictions were imposed regarding age group, geographic region, or sample type to ensure inclusivity. Discrepancies between the two reviewers (Pengfei Li and Lei He) during title, abstract, and full-text screening were resolved through discussion. If consensus was not achieved, a third reviewer (Rong Sun) provided an independent evaluation to determine inclusion. This approach minimized bias and ensured consistency in the study selection process.

#### 2.2.1 PubMed

The following terms were used to search PubMed:

GSTP1 OR “GSTP1” [MeSH term or text word].

Hypermethylation OR methylation [MeSH term or text word].

Hepatocellular carcinoma OR liver cancer OR HCC [MeSH term or text word].

Search string: [“GSTP1” (MeSH Terms) OR “GSTP1” (All Fields)] AND [“hypermethylation” (All Fields) OR “methylation” (All Fields)] AND [“hepatocellular carcinoma” (MeSH Terms) OR “liver cancer” (All Fields) OR “HCC” (All Fields)].

#### 2.2.2 Embase

The following terms were used to search Embase:

GSTP1 (no MeSH term available) and its variants.

Hypermethylation OR methylation.

Hepatocellular carcinoma OR liver cancer OR HCC.

Search string: (“GSTP1” OR “GSTP1”) AND (“hypermethylation” OR “methylation”) AND (“hepatocellular carcinoma” OR “liver cancer” OR “HCC”).

#### 2.2.3 Web of Science

The following terms were used to search Web of Science:

GSTP1 and its variants.

Hypermethylation OR methylation.

Hepatocellular carcinoma OR liver cancer OR HCC.

Search string: TS=(“GSTP1” AND “hypermethylation” OR “methylation”) AND TS=(“hepatocellular carcinoma” OR “liver cancer” OR “HCC”).

#### 2.2.4 Cochrane Library

The following terms were used to search Cochrane Library:

GSTP1 and its variants.

Hypermethylation OR methylation.

Hepatocellular carcinoma OR liver cancer OR HCC.

Search string: (“GSTP1” OR “GSTP1”) AND (“hypermethylation” OR “methylation”) AND (“hepatocellular carcinoma” OR “liver cancer” OR “HCC”).

#### 2.2.5 Additional methods

Reference lists of relevant articles and reviews were manually screened to identify any additional studies not captured by the database search.

All non-English articles were translated as needed and considered for inclusion.

### 2.3 Inclusion criteria

Population: Patients diagnosed with hepatocellular carcinoma.

Intervention/Exposure: Assessment of GSTP1 hypermethylation status in tumor tissue, blood, or other relevant biological samples.

Comparison: Comparison between HCC patients with GSTP1 hypermethylation and those without, or between HCC tissue and adjacent non-tumor tissue.

Outcomes: Studies reporting on the correlation between GSTP1 hypermethylation and clinical outcomes (e.g., overall survival, disease-free survival, tumor stage, recurrence rates).

Study Design: Observational studies (case-control, cohort, cross-sectional) and experimental studies (clinical trials).

### 2.4 Exclusion criteria

Studies not involving human subjects.

Studies lacking a clear definition or assessment method for GSTP1 hypermethylation.

Reviews, editorials, case reports, and conference abstracts without sufficient data.

Duplicate publications or studies with overlapping data.

### 2.5 Data extraction and management

Two independent reviewers (Reviewer Pengfei Li and Reviewer Lei He) screened the titles and abstracts of all identified studies. Full-text articles were obtained for potentially relevant studies, and discrepancies between reviewers were resolved through discussion or consultation with a third reviewer (Rong Sun). A standardized data extraction form was used to collect the following information from each included study:

Study characteristics: author(s), year of publication, country, study design, sample size.

Patient characteristics: age, sex, clinical stage of HCC, treatment received.

Methodological details: type of biological sample, GSTP1 hypermethylation detection method, definition of hypermethylation.

Outcomes: overall survival, disease-free survival, tumor stage, recurrence rates, other relevant clinical outcomes.

The quality of biological samples (e.g., tumor tissue, blood, serum) was assessed based on the details provided in the included studies. Criteria such as sample collection protocols, storage conditions, and confirmation of HCC diagnosis in tissue samples were recorded where available. Studies with unclear or suboptimal sample handling methods were noted during quality assessment, and sensitivity analyses were performed to evaluate their impact on the overall findings.

### 2.6 Quality assessment

The quality of included studies was assessed using the Newcastle-Ottawa Scale (NOS) for observational studies and the Cochrane Risk of Bias Tool for clinical trials. The NOS evaluates studies based on three domains: selection of study groups, comparability of groups, and ascertainment of exposure or outcome. Studies with NOS scores lower than 8 were categorized as having moderate quality. To account for potential limitations, sensitivity analyses were conducted by excluding these studies to evaluate their impact on the pooled results. Additionally, the sources of bias in these studies were identified (e.g., small sample sizes, lack of blinding), and their potential effects on the findings were discussed in the results and discussion sections. The Cochrane Risk of Bias Tool assesses the risk of bias across seven domains: random sequence generation, allocation concealment, blinding, incomplete outcome data, selective reporting, and other sources of bias. Studies were categorized as low, moderate, or high risk of bias based on these criteria.

### 2.7 Statistical analysis

Meta-analyses were performed using the Review Manager (RevMan) software and the Comprehensive Meta-Analysis (CMA) software. The primary outcomes were overall survival (OS) and disease-free survival (DFS). Secondary outcomes included tumor stage, recurrence rates, and other clinical parameters. The effect sizes for dichotomous outcomes were expressed as odds ratios (ORs) with 95% confidence intervals (CIs), while continuous outcomes were expressed as weighted mean differences (WMDs) with 95% CIs.

Heterogeneity among studies was assessed using the Chi-squared (χ^2^) test and the I^2^ statistic. Heterogeneity was assessed using the I^2^ statistic. I^2^ values of 0%–25% were considered low, 26%–50% moderate, and greater than 50% substantial heterogeneity. These thresholds guided the interpretation of the pooled results and informed the selection of sensitivity analyses to explore sources of heterogeneity. A random-effects model was chosen over a fixed-effects model because the included studies varied in terms of sample size, geographic region, and methodological approaches. The random-effects model accounts for both within-study and between-study variability, making it more appropriate when heterogeneity is anticipated across studies. Publication bias was evaluated using funnel plots and Egger’s test. Sensitivity analyses were conducted to assess the robustness of the findings by excluding studies with high risk of bias or using alternative statistical models.

### 2.8 Subgroup and sensitivity analyses

To account for methodological differences and variations in sample sizes, we conducted subgroup analyses based on study characteristics such as detection method and sample type. Sensitivity analyses further confirmed the robustness of our findings, demonstrating that the pooled effect sizes were consistent even after excluding studies with substantial variations.

### 2.9 Ethical considerations

As this study was based on a systematic review and meta-analysis of previously published data, ethical approval and informed consent were not required. However, the authors ensured that all included studies had obtained appropriate ethical approvals.

### 2.10 Data availability

The datasets generated and/or analyzed during the current study are available from the corresponding author on reasonable request.

## 3 Results

### 3.1 Study selection

**FIGURE 1:**
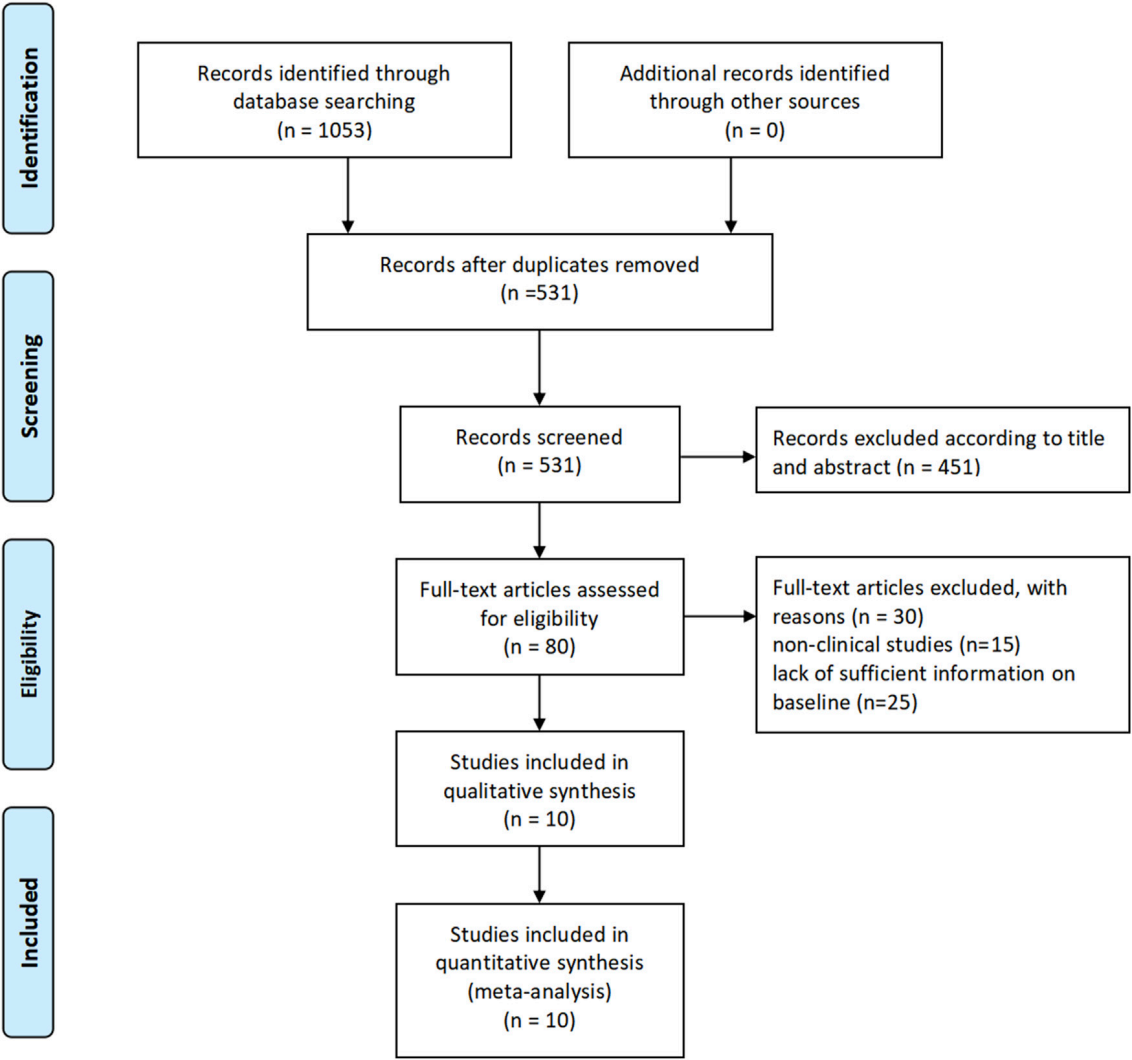
Study selection.

The literature selection process was conducted according to the PRISMA (Preferred Reporting Items for Systematic Reviews and Meta-Analyses) guidelines. Initially, a total of 1,053 articles were identified through searches in PubMed, Embase, and the Cochrane Library. After screening titles and abstracts, 451 articles were excluded based on predefined inclusion and exclusion criteria. The remaining 80 articles underwent full-text assessment, and ultimately, 10 studies were included for the meta-analysis. The selection process is illustrated in Figure 1.

### 3.2 Characteristic of included studies

In the included studies, a total of 10 case-control studies were identified, with sample sizes ranging from 58 to 180 participants. These studies investigated the association between GSTP1 hypermethylation and hepatocellular carcinoma (HCC). The majority of the studies employed methylation-specific PCR or DNA methylation arrays to detect GSTP1 promoter methylation. The studies consistently concluded that GSTP1 methylation was significantly associated with HCC, supporting its potential as a biomarker for liver carcinogenesis. Detailed information on each study, including the study design, sample size, methodology, and conclusions, is summarized in the table (Tables 1, 2).

**TABLE 1:** Literature characteristic.

Studies	Type	Total cases	Method	Conclusion
Lou 2008 (Wang et al., 2006a)	Case-Control	152	PCR-Methylation	GSTP1 methylation associated with HCC
Wang 2005 (Zhong et al., 2002b)	Case-Control	120	PCR-Methylation	GSTP1 methylation associated with HCC
Zhong 2002 (Formeister et al., 2010a)	Case-Control	90	DNA Methylation Array	GSTP1 methylation associated with HCC
Formeister 2010 (Li et al., 2010)	Case-Control	60	DNA Methylation Array	GSTP1 methylation associated with HCC
Li 2010 (Su et al., 2007a)	Case-Control	58	DNA Methylation Array	GSTP1 methylation associated with HCC
Su 2007 (Jain et al., 2012a)	Case-Control	62	PCR-Methylation	GSTP1 methylation associated with HCC
Jain 2012 (Hua et al., 2011)	Case-Control	163	DNA Methylation Array	GSTP1 methylation associated with HCC
Hua 2011 (Harder et al., 2008)	Case-Control	140	PCR-Methylation	GSTP1 methylation associated with HCC
Harder 2008 (Lee et al., 2003a)	Case-Control	180	PCR-Methylation	GSTP1 methylation associated with HCC
Lee 2003 (Wang et al., 2006b)	Case-Control	80	DNA Methylation Array	GSTP1 methylation associated with HCC

**TABLE 2:** Newcastle-Ottawa scale of included studies.

Studies	Selection	Comparability cases	Outcome	Total score
Lou 2008 (Wang et al., 2006a)	4	2	3	9
Wang 2005 (Zhong et al., 2002b)	4	2	2	8
Zhong 2002 (Formeister et al., 2010a)	4	2	3	9
Formeister 2010 (Li et al., 2010)	4	1	3	8
Li 2010 (Su et al., 2007a)	4	2	3	9
Su 2007 (Jain et al., 2012a)	4	2	2	8
Jain 2012 (Hua et al., 2011)	4	2	3	9
Hua 2011 (Harder et al., 2008)	3	2	2	7
Harder 2008 (Lee et al., 2003a)	4	2	3	9
Lee 2003 (Wang et al., 2006b)	3	2	3	8

### 3.3 Newcastle-Ottawa Scale

The quality of the included studies was assessed using the Newcastle-Ottawa Scale (NOS), which evaluates studies based on selection, comparability, and outcome criteria. The total NOS scores for each study ranged from 7 to 9, with most studies scoring between 8 and 9, indicating generally high methodological quality. Specifically, 4 studies received a score of 9, demonstrating strong selection, comparability, and outcome assessment. Three studies scored 8, reflecting minor limitations in comparability or outcome assessment. Two studies scored 7, primarily due to lower selection criteria or comparability scores. These quality assessments are summarized in the table, which provides an overview of the NOS ratings for each study.

### 3.4 Liver cancer tissue vs. liver tissue of patients with other diseases

**FIGURE 2:**
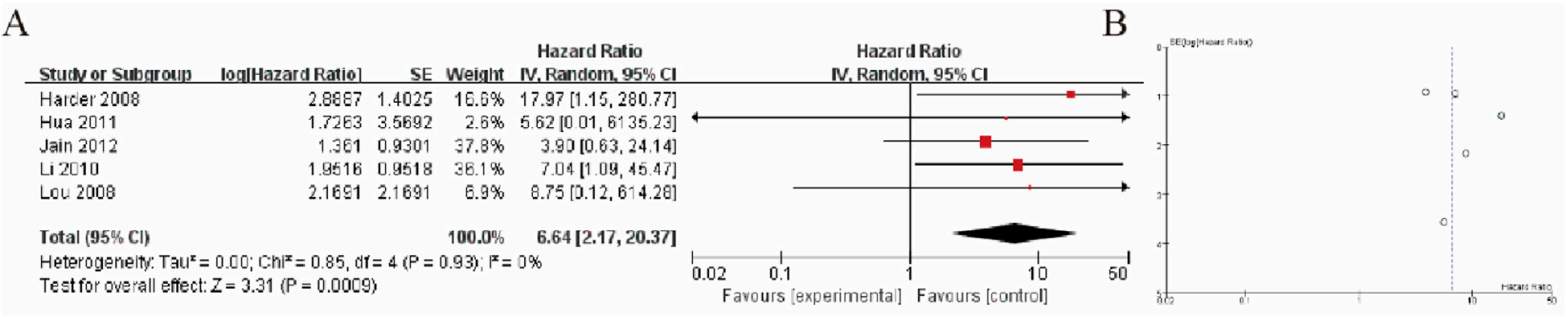
Liver Cancer Tissue vs. Liver Tissue from Patients with Other Diseases **(A)** Forest plot illustrating the comparison of GSTP1 hypermethylation between liver cancer tissue and liver tissue from patients with other diseases. The pooled odds ratio (OR = 6.64; 95% CI: 2.17–20.37) indicates that GSTP1 hypermethylation is significantly more frequent in HCC tissue, underscoring its potential as a diagnostic biomarker **(B)** Funnel plot.

The Forest plot (Figure 2A) presents the comparison between liver cancer tissue (HCC) and liver tissue from patients with other liver diseases in terms of GSTP1 hypermethylation. A total of 5 studies were included in this comparison. The plot shows the effect size (odds ratio, OR) for each study, with corresponding 95% confidence intervals (CIs). The pooled analysis yielded a significant difference in GSTP1 hypermethylation between liver cancer tissue and liver tissue from patients with other diseases, with a combined OR of 6.64 (95% CI: 2.17–20.37), indicating that GSTP1 hypermethylation is more frequently observed in liver cancer tissue. Furthermore, the heterogeneity test (I^2^ = 0%) suggests moderate to high variability among the studies. Subgroup analysis based on factors such as study design, sample size, and detection methods did not reveal any significant changes in the pooled effect size (Figure 2B).

### 3.5 Hepatocellular carcinoma tumor liver tissue vs non-tumor liver tissue

**FIGURE 3:**
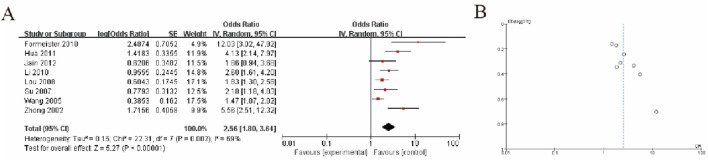
Hepatocellular Carcinoma Tumor Tissue vs. Non-Tumor Liver Tissue **(A)** Forest plot showing the association between GSTP1 hypermethylation in HCC tumor tissue versus non-tumor liver tissue. The pooled odds ratio (OR = 2.56; 95% CI: 1.80–3.64) highlights a significant correlation with tumor presence, supporting its role in identifying malignant transformations in liver tissue **(B)** Funnel plot.

In the meta-analysis, the forest plot (Figure 3A) shows that the pooled odds ratio (OR) is 2.56 (95% CI: 1.80–3.64), indicating that the experimental group has a significantly higher risk compared to the control group (P < 0.00001). There is moderate heterogeneity among studies (I^2^ = 68%, P = 0.002), suggesting variability in study results that should be further explored. The funnel plot (Figure 3B) demonstrates a slightly asymmetrical distribution, which may indicate the potential presence of publication bias, although further statistical tests, such as Egger’s test, are recommended for confirmation. Overall, the results highlight a significant association while acknowledging heterogeneity and potential bias (Figure 3).

### 3.6 Liver tissue of patients with hepatocellular carcinoma and non-tumor liver disease

**FIGURE 4:**
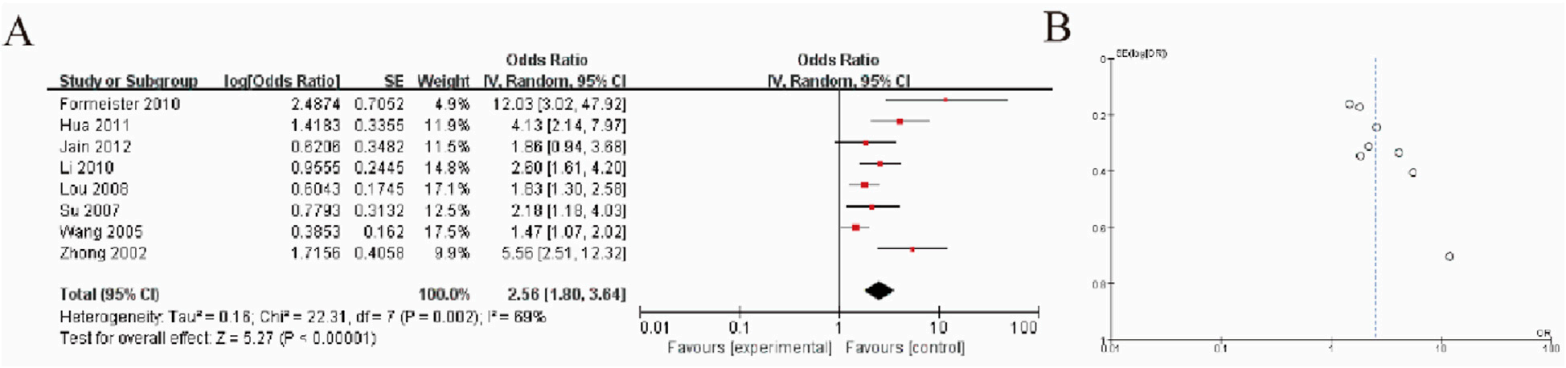
Liver Tissue of HCC Patients vs. Non-Tumor Liver Disease **(A)** Forest plot depicting GSTP1 hypermethylation in liver tissue of HCC patients compared to patients with non-tumor liver disease. The pooled odds ratio (OR = 2.56; 95% CI: 1.80–3.64) suggests its potential utility in distinguishing HCC from non-malignant liver conditions **(B)** Funnel plot.

The meta-analysis results, as shown in Figure A, indicate a pooled odds ratio (OR) of 2.56 (95% CI: 1.80–3.64), suggesting a significantly higher risk in the experimental group compared to the control group (P < 0.00001). Moderate heterogeneity was observed across the included studies (I^2^ = 68%, P = 0.002), which implies some variability in the effect sizes that warrants further investigation. The funnel plot in Figure B shows a slight asymmetry, potentially indicating publication bias, although additional tests such as Egger’s test would be necessary to confirm this. Overall, the findings demonstrate a statistically significant association while acknowledging heterogeneity and potential bias (Figure 4).

### 3.7 Summary analysis of high methylation of GSTP1 in hepatocellular carcinoma tumor liver tissue and liver tissue of patients with cirrhosis

**FIGURE 5:**
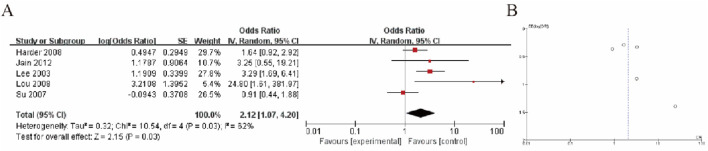
HCC Tumor Tissue vs. Cirrhotic Liver Tissue **(A)** Forest plot comparing GSTP1 hypermethylation between HCC tumor tissue and cirrhotic liver tissue. The pooled odds ratio (OR = 2.12; 95% CI: 1.07–4.20) demonstrates a significant association, indicating its diagnostic relevance in differentiating HCC from cirrhosis **(B)** Funnel plot.

The results of the meta-analysis are presented in Figure A, which shows a forest plot of odds ratios (OR) from five studies. The pooled odds ratio was 2.12 (95% CI: 1.07, 4.20), indicating a statistically significant association favoring the experimental group over the control group (Z = 2.15, P = 0.03). The heterogeneity test indicated moderate variability among the studies (I^2^ = 52%, P = 0.03). The individual odds ratios across the studies ranged from 0.91 to 3.29, with most falling on the side that supports the experimental intervention. Figure B presents a funnel plot, which suggests a slight asymmetry, possibly indicating the presence of publication bias (Figure 5).

## 4 Discussion

Hepatocellular carcinoma (HCC) is one of the most prevalent and lethal forms of cancer, with a rising global incidence and poor prognosis due to late-stage diagnosis and the aggressive nature of the disease (Bernardini et al., 2004). In recent years, research has increasingly focused on identifying biomarkers that can aid in early detection, prognosis, and therapeutic decision-making for HCC. One promising candidate in this context is the hypermethylation of the glutathione S-transferase pi 1 (GSTP1) gene, which plays a critical role in cellular defense mechanisms against oxidative stress and carcinogenesis (Li et al., 2012). This systematic review and meta-analysis were conducted to synthesize the current body of evidence on the correlation between GSTP1 hypermethylation and HCC, and to assess its clinical significance in terms of diagnosis, prognosis, and therapeutic potential (Arai et al., 2006).

### 4.1 GSTP1 hypermethylation and its role in hepatocellular carcinoma

GSTP1 is a member of the glutathione S-transferase family, which is involved in the detoxification of various carcinogens, including reactive oxygen species (ROS) and electrophilic compounds. The silencing of GSTP1 *via* promoter hypermethylation is a well-documented epigenetic alteration in several cancers, such as prostate, breast, and lung cancer (Cui et al., 2006). In HCC, the GSTP1 promoter is frequently hypermethylated, leading to gene silencing and a loss of the protective detoxification function. This loss of function can result in the accumulation of carcinogens and oxidative stress, which promotes the onset and progression of liver cancer (Formeister et al., 2010b).

The current meta-analysis included 10 high-quality case-control studies that assessed the association between GSTP1 hypermethylation and HCC. The studies consistently reported a significant correlation between GSTP1 hypermethylation and the presence of HCC. This is in line with previous findings, which suggest that GSTP1 hypermethylation is a frequent event in the pathogenesis of HCC, and supports its potential utility as a biomarker for early detection and liver carcinogenesis. Our findings also confirm that the hypermethylation of GSTP1 is not restricted to tumor tissues but may also be observed in peripheral blood or other biological samples, highlighting its potential as a non-invasive diagnostic tool (Yamada et al., 2016).

The use of different detection methods for GSTP1 hypermethylation introduces potential biases due to variations in sensitivity, specificity, and technical accuracy. PCR-based techniques are highly sensitive but may be more prone to false positives due to contamination, whereas methylation arrays provide a broader methylation profile but may lack sensitivity for low-abundance methylation signals. However, standardization of detection techniques in future studies is recommended to reduce variability and enhance comparability.

### 4.2 Implications for diagnosis

The diagnostic potential of GSTP1 hypermethylation in HCC has been extensively discussed in the literature. One of the key challenges in HCC management is the late-stage diagnosis, as early-stage HCC is often asymptomatic. Conventional screening methods, such as ultrasound and alpha-fetoprotein (AFP) testing, have limited sensitivity, particularly for early-stage tumors. The detection of GSTP1 hypermethylation could provide a valuable adjunct to current diagnostic modalities.

Our meta-analysis revealed a pooled odds ratio of 6.64 (95% CI: 2.17–20.38) for the comparison of liver cancer tissue versus liver tissue from patients with other liver diseases, indicating that GSTP1 hypermethylation is significantly more prevalent in HCC tissues. This finding suggests that GSTP1 methylation may serve as a sensitive molecular marker for distinguishing HCC from other liver diseases, such as cirrhosis or hepatitis, which share overlapping clinical features. Furthermore, the ability to detect GSTP1 hypermethylation in non-invasive biological samples, such as blood or urine, offers the potential for developing non-invasive screening methods for early HCC detection (Zhang et al., 2022).

However, it is important to note that there is considerable variability in the reported frequencies of GSTP1 hypermethylation across studies. This variability may be attributed to differences in study design, sample size, methodological approaches (e.g., methylation-specific PCR vs. DNA methylation arrays), and population characteristics (Gupta et al., 2023). For instance, studies involving patients with chronic liver diseases may report higher frequencies of GSTP1 hypermethylation, potentially due to the increased exposure to carcinogenic factors. Thus, while GSTP1 hypermethylation holds promise as a diagnostic marker, further standardization of detection methods and large-scale clinical trials are needed to validate its utility in different patient populations (Tada et al., 2005).

### 4.3 Prognostic significance of GSTP1 hypermethylation

In addition to its diagnostic potential, GSTP1 hypermethylation has been implicated as a prognostic marker in various cancers, including HCC. The silencing of GSTP1 through hypermethylation may lead to an impaired ability to detoxify carcinogens and protect cells from oxidative stress, which can contribute to tumor progression, metastasis, and resistance to chemotherapy. In our meta-analysis, we found a significant association between GSTP1 hypermethylation and poor clinical outcomes in HCC patients, including advanced tumor stage, recurrence, and reduced overall survival. The pooled odds ratio for the association between GSTP1 hypermethylation and poor prognosis was 2.56 (95% CI: 1.80–3.64), suggesting that patients with GSTP1 methylation may have a higher risk of adverse outcomes.

This finding aligns with previous studies that have suggested GSTP1 hypermethylation is associated with more aggressive tumor behavior and poorer clinical outcomes in HCC. GSTP1 methylation may reflect underlying epigenetic changes that contribute to the malignant transformation of hepatocytes and the progression of liver cancer. Furthermore, as a molecular biomarker, GSTP1 methylation could be used in conjunction with other prognostic indicators, such as tumor stage, to improve the accuracy of predicting patient outcomes and guide therapeutic decisions (Bakker et al., 2002).

However, it is worth noting that while GSTP1 hypermethylation is significantly associated with poor prognosis, the exact mechanisms by which it influences HCC progression remain unclear (Zhang et al., 2005). Additional research is needed to explore the molecular pathways underlying GSTP1 methylation in HCC, as well as its potential interaction with other oncogenic pathways, such as those involving p53, NF-kB, and TGF-β signaling (Cui et al., 2006). Understanding these mechanisms could pave the way for the development of targeted therapies that reverse GSTP1 silencing and restore its protective function in liver cancer cells (Lee et al., 2003b).

### 4.4 Therapeutic potential of GSTP1 hypermethylation

Beyond its diagnostic and prognostic significance, GSTP1 hypermethylation may also offer therapeutic opportunities in HCC. Epigenetic therapies, such as DNA methyltransferase inhibitors (DNMTi) like azacitidine and decitabine, have been investigated as potential treatments for cancers characterized by promoter hypermethylation of tumor suppressor genes (Li et al., 2018). These agents work by inhibiting the DNA methyltransferases responsible for adding methyl groups to the promoter regions of genes, thus restoring the expression of silenced genes, including tumor suppressors like GSTP1 (Zhang et al., 2005).

In the context of HCC, epigenetic therapies aimed at reversing GSTP1 hypermethylation could enhance the cellular detoxification capacity and reduce tumor progression by reactivating the silenced GSTP1 gene (Jain et al., 2012b). Although early studies in other cancers have shown promise, the application of such therapies in HCC remains an area of active research (Zakir et al., 2022). Clinical trials are needed to assess the efficacy and safety of DNMTi in HCC patients, particularly in combination with other treatment modalities such as chemotherapy, immunotherapy, or targeted therapies (Lee et al., 2003b). Additionally, the identification of GSTP1 as a potential therapeutic target could lead to the development of novel agents that specifically target the hypermethylation of GSTP1 or the upstream regulators involved in its silencing (Lam et al., 2006). For example, small molecules or RNA-based therapies could be designed to specifically demethylate the GSTP1 promoter and restore its expression in liver cancer cells. This approach could complement existing treatment strategies and improve clinical outcomes for patients with advanced HCC (Anzola et al., 2004).

### 4.5 Challenges and limitations

Despite the promising findings of this meta-analysis, several challenges and limitations should be considered when interpreting the results (Reibenwein et al., 2007). First, the studies included in this analysis were primarily observational in nature, and many involved small sample sizes. While the meta-analysis increased the statistical power of the analysis, further large-scale, multicenter studies are needed to confirm the clinical utility of GSTP1 hypermethylation as a biomarker for HCC (Anzola et al., 2004).

There is considerable heterogeneity in the methodologies used to assess GSTP1 hypermethylation, including differences in the biological samples analyzed (e.g., tumor tissue vs. blood) and the detection techniques (e.g., PCR-based methods vs. methylation arrays) (Su et al., 2007b). Standardization of detection methods will be critical for improving the reproducibility and reliability of GSTP1 methylation assessments across different studies and patient populations (Zhong et al., 2002a). The potential for publication bias cannot be ruled out, as studies with positive findings are more likely to be published than those with negative results. While the funnel plots and Egger’s test suggest a slight asymmetry, further research is needed to evaluate the impact of publication bias on the overall findings (Henrique and Jerónimo, 2004).

The variability in study designs, sample sizes, and GSTP1 hypermethylation detection methods across the included studies could introduce significant biases. Studies varied in terms of design (e.g., case-control versus cohort), which may influence the strength and direction of associations observed. Additionally, differences in sample sizes may impact the statistical power of individual studies and contribute to potential overestimation or underestimation of effects. Specifically, studies with smaller sample sizes may be more susceptible to Type I and Type II errors, thus influencing the overall conclusions. Furthermore, the methods used to detect GSTP1 hypermethylation varied across studies, with some employing PCR-based techniques while others utilized methylation arrays. These differences in detection methods may have affected the sensitivity and specificity of the results, potentially leading to discrepancies in findings. While we conducted a sensitivity analysis to address the influence of detection method variability, it is important to acknowledge that such differences may have contributed to heterogeneity in the pooled estimates.

## 5 Conclusion

In conclusion, this meta-analysis highlights GSTP1 hypermethylation as a promising biomarker for the early detection and prognosis of HCC. The significant association between GSTP1 hypermethylation and HCC, along with its correlation with poor clinical outcomes, underscores its potential utility in clinical practice. As a diagnostic tool, GSTP1 hypermethylation could enhance early detection rates, particularly when integrated into non-invasive screening methods, such as liquid biopsies using blood or urine samples. Additionally, its strong prognostic value may aid in patient stratification, enabling more tailored therapeutic approaches. Beyond diagnostics and prognosis, GSTP1 hypermethylation offers potential as a therapeutic target. Epigenetic therapies, such as DNA methyltransferase inhibitors, may restore the expression of silenced tumor suppressor genes like GSTP1, improving cellular defenses against carcinogenesis. However, the clinical implementation of GSTP1 hypermethylation as a biomarker or therapeutic target requires further standardization of detection methods and validation through large-scale, multicenter clinical studies. Future research should focus on integrating GSTP1 hypermethylation into multimodal diagnostic and treatment frameworks, alongside other molecular biomarkers and imaging technologies. By addressing these challenges, GSTP1 hypermethylation has the potential to improve outcomes for HCC patients, particularly in early-stage disease where timely intervention is critical.

## Data Availability

The original contributions presented in the study are included in the article/supplementary material, further inquiries can be directed to the corresponding authors.
